# Genome Sequence of a Novel, Newly Identified Isolate of Guinea Pig Cytomegalovirus, the CIDMTR Strain

**DOI:** 10.1128/genomeA.01052-13

**Published:** 2013-12-26

**Authors:** Mark R. Schleiss, Nelmary Hernandez-Alvarado, Thiruvarangan Ramaraj, John A. Crow

**Affiliations:** University of Minnesota Medical School, Department of Pediatrics, Division of Pediatric Infectious Diseases and Immunology, Minneapolis, Minnesota, USAa; National Center for Genome Resources, Santa Fe, New Mexico, USAb

## Abstract

The sequence of a newly discovered isolate of guinea pig cytomegalovirus (GPCMV), the CIDMTR strain, was determined. The 232,778-nucleotide genome was generally well conserved with that of the 22122 reference strain, although some regions of substantial sequence divergence allowed annotation of strain-specific open reading frames encoding putative immune modulation gene products.

## GENOME ANNOUNCEMENT

Guinea pig cytomegalovirus (GPCMV) provides a useful model for congenital human cytomegalovirus (HCMV) infection (1, 2). GPCMV strain 22122, isolated by Hartley in 1957 (ATCC VR-682), has to date been the only strain available (3). This strain was sequenced using DNA purified both from tissue culture (TC)-passaged virus (4) and from guinea pig salivary gland (SG) homogenates following *in vivo* passage, with only minor differences noted (5). Some genomic instability of strain 22122 has been noted (6, 7), and its isolation and passage history are incompletely defined. Therefore, isolation of additional strains is desirable and would facilitate the modeling of HCMV reinfection, an emerging concern in women (8, 9).

Commercially purchased Hartley strain guinea pigs were found to be GPCMV seropositive. A seropositive animal was immunosuppressed with cyclophosphamide, 100 mg/kg (10), and sacrificed 7 days later. SG homogenate was passaged *in vivo* in seronegative strain 2 guinea pigs. SG homogenates from these animals were then passaged on guinea pig fibroblasts, yielding plaques compatible with GPCMV. Following two additional passages in TC, virions were purified, and lysis buffer (200 mM NaCl, 2% SDS, and 200 µg/ml proteinase K in Tris-EDTA [TE]) was added. Following incubation at 68°C overnight, three phenol-chloroform extractions were performed, followed by ethanol precipitation of viral DNA.

Genomic sequencing was performed using Illumina MiSeq and Pacific Biosciences (PacBio) RS platforms. Approximately 5.2 million 151-bp paired-end MiSeq reads were generated at the UMN Biomedical Genomics Center with a nominal insert size of 400 bp. Removal of low-quality reads and phiX sequences resulted in a set of 4.0 million cleaned reads, approximately 11,000× coverage. Scaffolds were generated from the cleaned Illumina reads using the ABySS assembler ver. 1.3.4 (11), and quality was assessed by mapping reads back to the assembly and manual comparison with KC503762 (5, 12). Special attention was paid to correct alignment and orientation of paired ends. Regions of weak coverage and scaffold gaps were identified and closed by manual local assembly (13) or by Sanger sequencing. Independent validation of the pseudomolecule was performed using the longer PacBio RS reads. Single-molecule real-time (SMRT) sequencing produced 998 high-quality (“corrected”) reads ranging between 509 and 15,898 bp, median 6,257 bp (~27× coverage), as well as another set of scaffolds. These data were used to evaluate the Illumina assembly, specifically its structural correctness, and to correct misassembled repeat regions.

Comparison of the sequence with 22122 revealed a region of discrepancy spanning nucleotides ~198780 to 201300 of the CIDMTR sequence. A deletion of approximately 1.5 kb in this region resulted in the loss of a gp138 gene family member that was previously annotated in 22122 (5). A second region spanning coordinates 221540 through 224780 contained 1.45 kb of additional sequence that was not present in 22122, including a novel copy of a major histocompatibility complex (MHC) homolog, annotated as gp147.1 (14). Similar to HCMV, this region of the GPCMV genome appears prone to deletions, gene duplications, and rearrangements (15). Additional sequence analysis of wild-type strains may help resolve which sequence features represent the most authentic GPCMV genome.

### Nucleotide sequence accession number.

The draft genome sequence for this strain has been deposited with the EMBL Nucleotide Sequence Database under the accession number HG531783. The CIDMTR strain of GPCMV has been deposited with the American Type Culture Collection (Manassas, VA) and is available from the corresponding author.
